# Real-time images of tidal recruitment using lung ultrasound

**DOI:** 10.1186/s13089-015-0036-2

**Published:** 2015-12-12

**Authors:** Gerardo Tusman, Cecilia M. Acosta, Marco Nicola, Mariano Esperatti, Stephan H. Bohm, Fernando Suarez-Sipmann

**Affiliations:** Department of Anesthesia, Hospital Privado de Comunidad, Córdoba 4545, 7600 Mar del Plata, Buenos Aires Argentina; Intensive Care Medicine, Hospital Privado de Comunidad, Mar del Plata, Buenos Aires Argentina; Swisstom AG, Landquart, Switzerland; Department of Surgical Sciences, Section of Anesthesia and Critical Care Hedenstierna Laboratory, Uppsala University Hospital, Uppsala, Sweden; CIBER de Enfermedades Respiratorias, Instituto de Salud Carlos III, Madrid, Spain

**Keywords:** Tidal recruitment, Atelectasis, VILI, Lung ultrasound, Recruitment maneuvers

## Abstract

**Background:**

Ventilator-induced lung injury is a form of mechanical damage leading to a pulmonary inflammatory response related to the use of mechanical ventilation enhanced by the presence of atelectasis. One proposed mechanism of this injury is the repetitive opening and closing of collapsed alveoli and small airways within these atelectatic areas—a phenomenon called tidal recruitment. The presence of tidal recruitment is difficult to detect, even with high-resolution images of the lungs like CT scan. The purpose of this article is to give evidence of tidal recruitment by lung ultrasound.

**Findings:**

A standard lung ultrasound inspection detected lung zones of atelectasis in mechanically ventilated patients. With a linear probe placed in the intercostal oblique position. We observed tidal recruitment within atelectasis as an improvement in aeration at the end of inspiration followed by the re-collapse at the end of expiration. This mechanism disappeared after the performance of a lung recruitment maneuver.

**Conclusions:**

Lung ultrasound was helpful in detecting the presence of atelectasis and tidal recruitment and in confirming their resolution after a lung recruitment maneuver.

**Electronic supplementary material:**

The online version of this article (doi:10.1186/s13089-015-0036-2) contains supplementary material, which is available to authorized users.

## Background

Tidal recruitment (TR) is the repetitive opening and closing of collapsed alveoli during the mechanical respiratory cycle [1, 2]. The resultant high tissue stress in these atelectatic zones, and especially in the boundary areas between collapsed and open lung areas, can trigger a local inflammatory response and injure the alveolar–capillary membrane [3]. This is one of the proposed mechanisms of ventilator-induced lung injury (VILI) that impairs the outcome of patients with acute respiratory distress syndrome [1–4]. The other mechanism of VILI is tidal overinflation that could appear in some ventral areas of the lungs at the end of inspiration [5].

Nowadays, there is clear evidence that VILI can affect even patients with previous healthy lungs, the common scenario during general anesthesia [6–8]. This is of special relevance in surgical patients since there is an established link between atelectasis, VILI and the development of post-operative pulmonary complications [8–10].

The use of a lung protective ventilatory strategy resulted in a significant decrease in mortality in ARDS patients by the reduction of VILI during mechanical ventilation [4]. This ventilatory strategy minimized cyclic overdistensión and TR thereby decreasing the mechanical stress on lung tissue using low tidal volumes, plateau pressures and positive end-expiratory pressure (PEEP). However, this ventilation strategy does not fully eliminate TR because of the persistence of atelectasis along the mechanical ventilation period. Contrarily, lung recruitment—i.e. a ventilatory strategy that reverts atelectasis by applying a few mechanical breaths at high airways pressure—can potentially avoid the occurrence of VILI by TR [8, 11, 12]. This is simply because, by definition, TR is not possible without the presence of atelectasis.

Tidal recruitment is difficult to detect clinically and experimentally without the use of advanced imaging techniques. Currently, high-resolution CT scan is considered the gold standard method to detect TR in atelectatic areas by measuring the difference in aeration observed between static images taken at end-inspiration and end-expiration [12]. However, this method is expensive, time consuming; it submits the patient to radiation and cannot be applied at the bedside.

Lung ultrasound (LUS) is an attractive option to see TR in mechanical ventilated patients because, as opposed to CT, it is non-invasive, radiation-free and simple to use at the bedside [13, 14]. It has a high sensitivity and specificity for detecting lung collapse which appears as a LUS consolidation pattern [13, 15]. The real-time nature of LUS images of atelectasis constitutes an important feature to detect this dynamic mechanism of VILI during the respiratory cycle. This short communication describes the use of LUS images to detect TR in mechanically ventilated patients during anesthesia and its resolution after applying a lung recruitment maneuver.

### LUS technique to assess TR

LUS assessment was performed with the portable echograph MicroMax (Sonosite, Bothell, WA, USA) using a linear probe of 6–12 MHz. The echo probe placed in the longitudinal position along the ribs allowing for a systematic inspection of all lung areas [13, 15]. Once atelectasis areas were localized, two LUS’s technical factors were crucial to detect TR in our patients. One factor was the use of a linear 6–12 MHz probe which provides high-resolution images of atelectasis. The routine use of a linear probe is uncommon for lung studies in adult patients because it reaches a maximum depth of only 6 cm. However, 6 cm of depth is enough to obtain a good view of the sub-pleural condensations with even more detail (figures and videos) [15, 16].

The other factor was to place the probe in the oblique position between the ribs to get a complete vision of the atelectatic area during the whole respiratory cycle [15]. Contrarily, applying the classical longitudinal approach (the “bat sign”) could hide parts of atelectasis below the acoustic shadow of the ribs during the respiratory cycle, thus diminishing the view of the TR phenomenon.

### Clinical evidence of TR and its treatment by recruitment maneuver

We present two clinical examples of how LUS easily detects TR and how lung recruitment maneuvers effectively eliminate such mechanism of VILI during anesthesia.

**Fig. 1:**
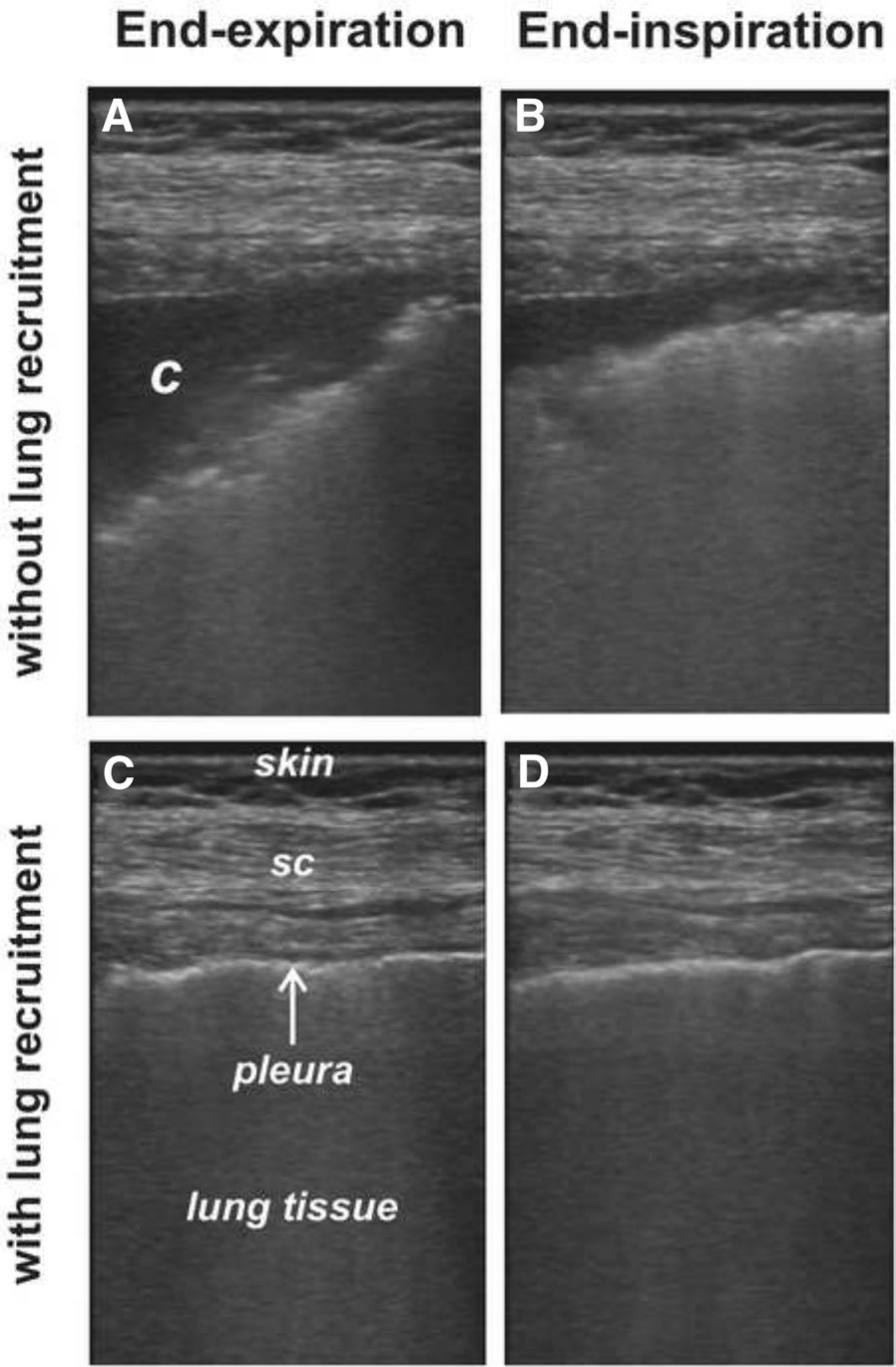
Tidal recruitment in an anesthetized adult. All ultrasound images were obtained at the same lung region. *Figures* show atelectasis and tidal recruitment despite a protective ventilation pattern (**A**, **B**). A pulmonary consolidation (*c*) or atelectasis appears as a hypoechoic area mixed with airway bronchograms. Visual differences in the degree of lung aeration and the area of atelectasis between end-expiration and end-inspiration define the presence of tidal recruitment. **C** and **D** were obtained after a lung recruitment maneuver. They represent almost normal lung ultrasound images during end-expiration and end-inspiration, with the typical structures of skin, sub-cutaneous tissue (*sc*), pleural line and lung tissue. No differences in aeration were found during the breathing cycle

Figure 1 and Additional file 1: video S1 belong to a 68-year-old woman scheduled for surgical repair of multiple leg fractures. After induction of general anesthesia, protective ventilation was applied using volume-controlled ventilation [4, 6, 10]. This consisted of a tidal volume of 6 mL/kg ideal body weight, respiratory rate of 16 bpm, I:E ratio of 1:2, PEEP of 8 cmH_2_O, and FIO_2_ of 0.6. The corresponding plateau pressure was 27 cmH_2_O and the finger pulse oximetry displayed a value of 94 %.

Ten minutes later LUS confirmed bilateral atelectasis in the most dependent lung areas, with the probe placed within the intercostal space at the posterior axillary line just above the diaphragm. This corresponds to the usual distribution of atelectasis during general anesthesia [15, 18]. Most of the observed atelectatic area present at end-expiration (Fig. 1A) disappeared (i.e. was tidally recruited) at the end of the next inspiration (Fig. 1B; Additional file 1: video S1).

We then performed a lung recruitment maneuver according to the method described by our group [19]. This ventilatory strategy consisted of a step-wise increment in airway pressure to reach the pressure that re-expands atelectasis. The response to lung recruitment was confirmed by the complete resolution of atelectasis observed in LUS images of the most dependent areas of the lungs [15, 17]. Later on, a step-wise decrement in PEEP allowed the detection of the minimum level that prevents the reappearance of atelectasis in LUS images. After lung recruitment, ventilatory settings were returned to the protective ventilation pattern as described before, but this time maintaining the found PEEP level of 10 cmH_2_O to maintain the lungs free of atelectasis. This ventilatory pattern resulted in a plateau pressure of 25 cmH_2_O and a pulse oximetry reading of 99 % using a FIO_2_ of 0.3. LUS no longer showed neither atelectasis nor tidal recruitment (Fig. 1C, D; Additional file 1: video S1).

**Fig. 2:**
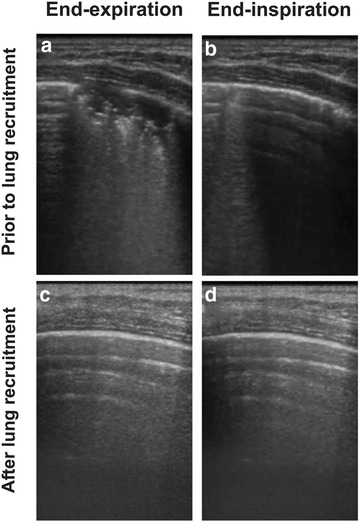
Tidal recruitment in an anesthetized child. All ultrasound images were obtained at the same lung region. **a,** **b** Atelectasis and tidal recruitment despite a protective ventilation pattern. **c,** **d** Normal LUS images after a lung recruitment maneuver

We observed similar findings in a healthy 3-year-old anesthetized child scheduled for laparoscopic surgery. Figure 2a, b shows atelectasis and TR in dependent, para-diaphragmatic lung areas using a protective ventilatory pattern. Figure 2c, d obtained after a lung recruitment maneuver confirms the absence of atelectasis and tidal recruitment showing normal lung images. Additional file 2: video S2 presents the dynamic sequence of the corresponding images before and after lung recruitment.

### Commentary

The role of LUS in detecting lung consolidation has been very well described and validated [13–15]. LUS is also useful to evaluate the effects of ventilatory strategies on atelectasis like PEEP or recruitment maneuvers [14, 15, 17, 20, 21]. Such evaluation is based on the degree of lung aeration observed in the ultrasound images. However, to our knowledge, the diagnosis of TR by LUS has not been described before.

The presented cases illustrate how the atelectatic area appearing as a LUS consolidation pattern decreased during inspiration and returned to the original size at the end of expiration, confirming the presence of this mechanism of VILI in healthy lung ventilated with recommended lung protective settings during anesthesia. Furthermore, the elimination of the consolidated lung area by means of a lung recruitment maneuver confirmed the diagnosis of atelectasis and the consequent mechanism of TR.

## Conclusions

Tidal recruitment is one of the principal described mechanisms producing VILI that affects mechanically ventilated patients with atelectasis. LUS can detect TR in an easy way by capturing its dynamic nature with real-time imaging. LUS also helped in confirming the resolution of atelectasis and TR after lung recruitment and an individualized PEEP re-adjustment and can therefore become a valuable monitoring tool to prevent VILI at the bedside.

## Consent

The institutional review board approved this publication and the corresponding written informed consent was obtained from the patient’s relatives.

## Additional files

10.1186/s13089-015-0036-2 This video shows the dynamic sequence corresponding to Fig. 1 in an anesthetized adult. Left: tidal recruitment can be seen during lung protective ventilation. Right: atelectasis and tidal recruitment disappeared after lung recruitment but using the same protective ventilatory pattern than before.
10.1186/s13089-015-0036-2 This video shows the dynamic sequence corresponding to Fig. 2 in an anesthetized child. Left: tidal recruitment before a lung recruitment maneuver. Right: images after lung recruitment.
